# UV Sensitivity of MOS Structures with Silicon Nanoclusters

**DOI:** 10.3390/s19102277

**Published:** 2019-05-17

**Authors:** Mario Curiel, Nicola Nedev, Judith Paz, Oscar Perez, Benjamin Valdez, David Mateos, Abraham Arias, Diana Nesheva, Emil Manolov, Roumen Nedev, Valeri Dzhurkov

**Affiliations:** 1 Universidad Autónoma de Baja California, Instituto de Ingeniería, Benito Juárez Blvd., s/n, 21280 Mexicali, Baja California, México; mcuriel@uabc.edu.mx (M.C.); judith.paz@uabc.edu.mx (J.P.); oscar.manuel.perez.landeros@uabc.edu.mx (O.P.); benval@uabc.edu.mx (B.V.); david.mateos@uabc.edu.mx (D.M.); 2 Universidad Autónoma de Baja California, Facultad de Ingeniería, Benito Juárez Blvd., s/n, 21280 Mexicali, Baja California, México; arias.abraham@uabc.edu.mx; 3 Institute of Solid State Physics, Bulgarian Academy of Sciences,72 Tzarigradsko Chaussee Blvd,1784 Sofia, Bulgaria; nesheva@issp.bas.bg (D.N.); e.manolov1@gmail.com (E.M.); valeri.dzhurkov@gmail.com (V.D.); 4 Universidad Politécnica de Baja California, Calle de la Claridad s/n, 21376 Mexicali, Baja California, México; rnikolovn@upbc.edu.mx

**Keywords:** UV, selective sensitivity, MOS, Si nanoclusters

## Abstract

Selective UV sensitivity was observed in Metal-Oxide-Semiconductor structures with Si nanoclusters. Si nanocrystals and amorphous Si nanoparticles (a-Si NPs) were obtained by furnace annealing of SiO_x_ films with x = 1.15 for 60 min in N_2_ at 1000 and 700 °C, respectively. XPS and TEM analysis prove phase separation and formation of Si nanocrystals in SiO_2_, while the a-Si NPs are formed in SiO_1.7_ matrix. Both types of structures show selective sensitivity to UV light; the effect is more pronounced in the structure with nanocrystals. The responsivity of the nanocrystal structure to 365 nm UV light is ~ 4 times higher than that to green light at 4 V applied to the top contact. The observed effect is explained by assuming that only short wavelength radiation generates photocarriers in the amorphous and crystalline nanoclusters.

## 1. Introduction

Metal-Oxide-Semiconductor (MOS) structures with silicon nanocrystals have been actively studied for electronic and optoelectronic applications in the past decades [1,2,3,4]. The processes of formation of silicon nanoclusters (Si NCs) in MOS structures are compatible with today’s microelectronic technology, which make them applicable in floating gate non-volatile memory devices [1,4], Si based light emitters [5,6] and “third generation” solar cells [7]. Thin SiO_x_ films containing amorphous and/or crystalline Si nanoclusters are promising for application in optoelectronic devices, because of quantum effects in the NCs. The electrical and optoelectronic properties of MIS structures with NCs depend strongly on the Si nanoparticle size, as well as on the structural and dielectric properties of the surrounding medium.

Semiconductor diodes have been widely used as sensors of ultraviolet (UV) light. UV enhanced silicon photodiodes are well-established devices for UV detection [8], although they have the drawback to respond to low energy radiation. Since the Si bandgap is ~1.1 eV these photodiodes require filters to block out the visible and infrared photons. Sheng et al. have published results for a visible blind Si based UV detector that uses down-shifting luminophore, which absorbs in the 250–360 nm range and emits at ~610 nm [9]. Recently, various wide gap semiconductors (E_g_ > 3.1 eV) have been studied for visible blind UV detectors. Some materials of interest are Al_x_Ga_1-x_N with composition varying between GaN and AlN [10,11], ZnO, NiO [12,13,14] and Ga_2_O_3_ [15]. The combination of p-NiO and n-ZnO has been used for fabrication of transparent photodiodes [13,14]. ZnO nanoparticles uniformly distributed on diatomite surface have shown strong enhancement of absorption and scattering coefficients in the 300–450 nm wavelength range [16,17]. Ga_2_O_3_ has a band gap close to 5 eV, which makes this material suitable for a solar-blind detector, with a cut-off below 280 nm [18,19,20].

Photoresponse of Metal-Oxide-Semiconductor structures with silicon nanoclusters has also been studied [21,22,23]. MOS photodiodes are attractive because they can be fabricated using standard processes for microelectronics. However, when considered for UV detection the MOS diodes suffer an important limitation, the main response is from visible light. In this work we present results for MOS structures with Si nanoclusters in the gate oxide with main response in the UV region, which depends on the nanocluster size.

## 2. Materials and Methods

Silicon oxide (SiO_x_) layers with x = 1.15 and nominal thickness of 40 nm were deposited on 4–6 Ω cm (100) n-type Si wafers by thermal evaporation of silicon monoxide. The evaporation was carried out at room temperature and a vacuum of 1 × 10^−3^ Pa. The film thickness and deposition rate were monitored using a quartz microbalance. Before the film depositions the silicon wafers were chemically cleaned using a standard procedure for the microelectronics industry. Three groups of samples were prepared: Annealed at 250 °C for 30 min in Ar (control samples); annealed at 700 °C to form amorphous silicon nanoclusters and annealed at 1000 °C to grow Si nanocrystals in the SiO_x_ layer [24,25]. The high temperature annealing was carried out in nitrogen for 60 min.

TEM analysis was performed using a Jeol JEM-2010 microscope operating at 200 kV. Samples for cross-sectional view micrographs were prepared by gluing two samples film to film and then ground and milled from both sides to electron transparency. XPS spectra were measured by SPECS high resolution spectrometer using Al Kα X-ray source (1486.6 eV).

MOS capacitors were formed for electrical characterization by evaporation through a mask of Al top and back contacts. The top contacts were with diameter of 0.5 mm, thickness of ~0.5 μm and distance between the centers of two circles of 1 mm. Current-Voltage (I-V) characteristics were measured using Semiconductor Characterization System Keithley 4200-SCS. The I-V dependencies were measured in dark and under illumination with red, green, blue, UV and warm light LED diodes. According to the specifications, the UV spectrum is centered at 365 nm, while the warm light LED spectrum is similar to black body radiation at 3000 K, which does not emit in the UV range. The optical power at the sample position was measured by Thorlabs S120C Standard Photodiode Power Sensor connected to PM100USB Optical Power and Energy Meter. The obtained values for the red, green, blue, UV and warm light LEDs were 2.23, 1.9, 2.4, 0.225 and 0.81 mW/cm^2^ respectively.

## 3. Results and Discussion

### 3.1. Compositional Characterization

**Figure 1:**
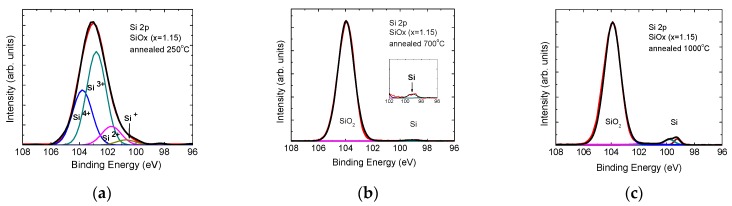
Si 2p core level spectra of a control SiO_x_ film (**a**) and of films annealed at 700 °C (**b**) and 1000 °C (**c**). The notations Si^+^, Si^2+^, Si^3+^ and Si^4+^ are used for the four oxidation states.

Figure 1 a–c show XPS spectra of the Si 2p core level of SiO_x_ films annealed at 250, 700 and 1000 °C, respectively. The control film spectrum (Figure 1a) shows contribution of the four oxidation states of Si, according to fitting with four Gaussians curves with maximums at 100.7 eV (Si^+^), 101.7 eV (Si^2+^), 102.8 eV (Si^3+^) and 103.8 eV (Si^4+^). The main contribution in the suboxide matrix is from Si_2_O_3_ and not from the stoichiometric one, SiO_2_. The XPS spectrum of the 700 °C annealed film (Figure 1b) differs from that of the control sample. The amount of excess Si atoms bonded to O in suboxides decreases significantly. A detailed observation of the 96–102 eV interval of binding energies reveals a small contribution of pure Si phase (inset). In the 1000 °C annealed sample (Figure 1c) only clearly separated peaks corresponding to stoichiometric SiO_2_ and pure Si phase are observed. The doublet Si 2p 3/2, Si 2p 1/2 with binding energies of 99.4 and 99.8 eV is also observed using Gaussian fitting of the pure Si peak. The XPS measurements indicate that the matrix in which the nanoclusters are formed is stoichiometric SiO_2_ and SiO_x_ with x = 1.7 after 1000 and 700 °C annealing, respectively.

### 3.2. Structural Characterization

**Figure 2:**
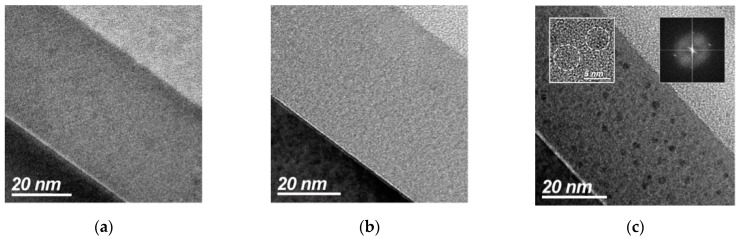
Cross-sectional TEM (XTEM) images of a SiO_x_ (1.15) control sample (**a**), and annealed at 700 °C (**b**) and 1000 °C (**c**) samples. The insets show a HR-TEM image of two crystals (left) and a Fast Fourier Transform of selected Si nanocrystal area (right).

Figure 2a–c show cross-sectional TEM (XTEM) images of a control SiO_x_ film, SiO_x_ films annealed at 700 °C and 1000 °C, respectively. The control structure shows a homogeneous contrast of SiO_x_ film (Figure 2a), while the 700 °C structure image exhibits a slight contrast variation related to the amorphous nature of the Si nanoclusters (Figure 2b). The 1000 °C structure shows dark clusters resulting from silicon aggregation (Figure 2c). High resolution TEM imaging on a single dark spot reveals a crystalline nature of the cluster (left inset in Figure 2c); a Fast Fourier Transform of the high resolution TEM (HR-TEM) image reveals a periodic arrangement inside the clusters (right inset in Figure 2c). The nanocrystal diameters are in the ~4–6 nm range; the nanocrystals are randomly distributed in an amorphous matrix all along the film thickness. This observation is in good agreement with previously reported results [24,25]. In the samples with high temperature annealing a thin SiO_2_ film (free of nanoclusters) of ~5 nm is seen at the film surface, most likely produced by air exposure, as has been previously reported [24].

### 3.3. Electrical Characterization

**Figure 3:**
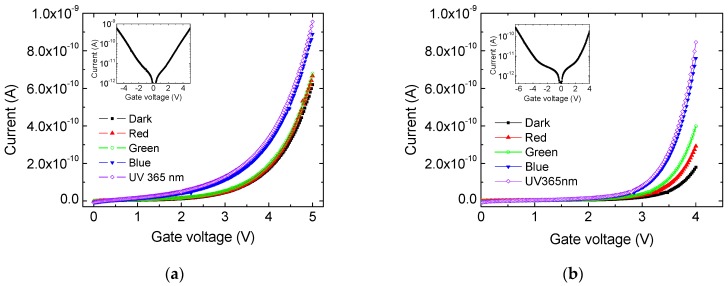
I-V dependencies of c-Si/SiO_x_/Al structures annealed at 700 (**a**) and 1000 ^o^C (**b**) in dark and under illumination with red, green, blue and UV light. The inset figures show dark I-V characteristics in semi-logarithmic scale measured in both directions.

Figure 3a,b display I-V dependencies of c-Si/SiO_x_/Al structures annealed at 700 and 1000 °C, respectively. The characteristics were measured in dark and under illumination with red, green, blue and UV light. The dark current at positive voltages is due to electrons injected from the Si substrate in the oxide, which then tunnel between neighboring nanoclusters towards the Al electrode. Almost symmetric characteristics were measured on the structure with a-Si nanoparticles (inset in Figure 3a), while in the structure with nanocrystals the current increases faster at positive voltages (inset in Figure 3b). However, the ratio of currents flowing under forward bias (positive voltage V_g_ on the top electrode for n-type Si substrate) and reverse bias at V_g_ = ±4 V is about one order of magnitude. This is an important difference between the nanocrystal structures studied here and the ones reported in [21,22] where the current in forward direction is much higher than in reverse direction, about two orders of magnitude or more. Another difference between the dark current dependences in the insets in Figure 3 is the voltage at which exponential current increase occurs. For the structure with a-Si particles it is about 2 V, while for the nanocrystal structure it is higher, ~3 and −3.5 V forward and reverse biases, respectively. This observation is in agreement with the XPS results, which show complete phase separation and formation of Si phase in stoichiometric SiO_2_ after annealing at 1000 °C. For both types of structures, the UV and blue lights cause significant increase of the current, while the green and red light lead to very small changes. It should be pointed out that the optical power of the blue LED is ~10.6 times higher than that of the UV LED, i.e., even the photoresponse to blue light is small compared to the UV response. An important difference between the I-V characteristics in Figure 3a,b is the higher current under red and green light illumination of the structure annealed at 1000 °C. The observed difference may be explained assuming that light generates photocarriers in nanoclusters outside of the top contact area, which contribute to the current through the dielectric. Part of the photogenerated carriers diffuse under the top contact due to concentration gradient and/or are driven below the contact by the electric field from distances outside the contact periphery, comparable to the layer thickness, ~40 nm. Further, these carriers are separated by the electric field and tunnel between neighboring clusters leading to an increase of the current through the MOS structure. The optical bandgap of the amorphous Si nanoclusters obtained after 700 °C annealing was estimated to be ~2.6 eV [26]. It may be assumed that the gap of the nanocrystals is ≤ 1.8 eV according to results published in [27] for nanocrystals with similar size. Thus, in amorphous nanoclusters light with wavelength (λ) close to or smaller than 480 nm can generate photocarriers, while for nanocrystals the value of λ is ≤ 690 nm.

**Figure 4:**
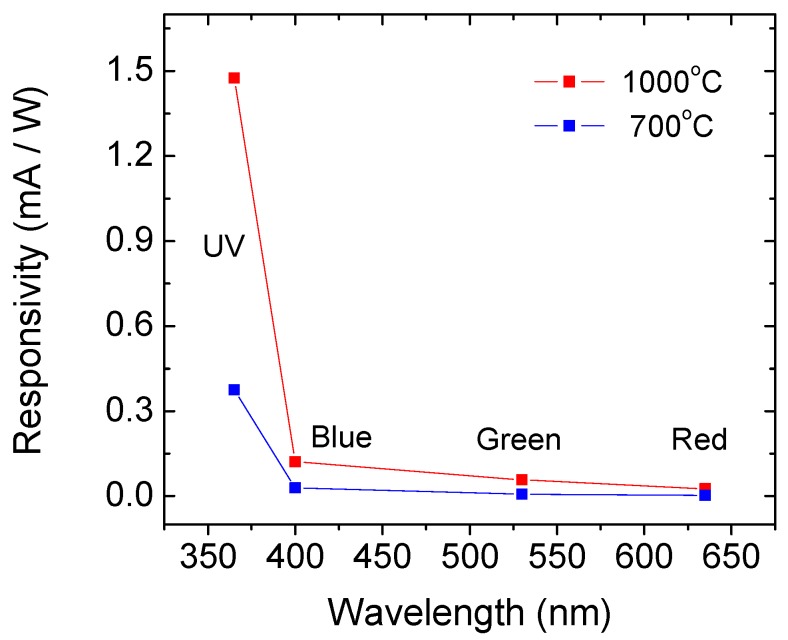
Responsivities at 4 V of structures with Si nanocrystals (red curve) and amorphous Si nanoparticles (blue curve).

The responsivity of the two types of structures at 4 V applied on the top contact is shown in Figure 4. It is clearly seen that the structure with nanocrystals show higher responsivity to UV light and is much more appropriate for application in UV sensors, though its response to green and red wavelengths is also higher. It should be pointed out that the real responsivity probably is higher than that in Figure 4, which was calculated using the top Al contact as active area. Since the Al contact is thick, the light does not penetrate below it. More likely the active area is much smaller, approximately 2πr ×film thickness, where r is the radius of the top contact. The obtained responsivity is much smaller than the observed giant UV photoresponse in GaN nanowire photodetector decorated with Pt nanoparticles [28], as well as of the GaN based photodiodes [10]. However, the proposed structure has the advantage to be based on Si technology, i.e., uses very well developed processes which result in devices with high reliability and allow large scale production at low cost. Other advantages of the studied MOS devices compared to similar nanocrystal structures are: Higher ratio between responses in the UV and the visible range, lower dark current, especially in forward direction and lower trap density in the layer with nanoclusters, especially for holes, in contrast to results in [21]. Trapped charge carriers may cause shift of the I-V characteristics in consecutive measurements.

**Figure 5:**
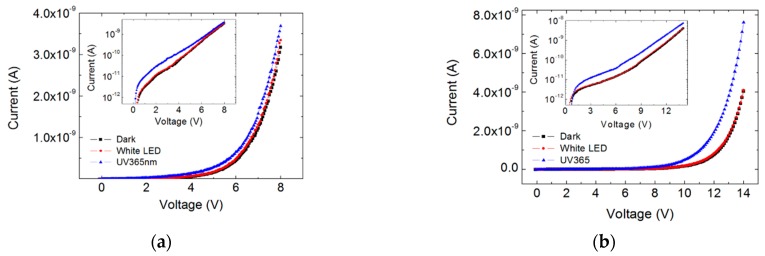
I-V dependencies in dark and under warm light and UV illumination measured in lateral configuration on samples annealed at 700 (**a**) and 1000 °C (**b**). The inset figures show the same dependencies in semi-logarithmic scale.

Figure 5a,b shows I-V dependencies in dark and under warm light and UV illumination measured in lateral configuration, i.e., between two top contacts, on samples annealed at 700 and 1000 °C. In this configuration the current flows from the negatively biased Al contact through the SiO_x_ layer, the Si substrate and again through SiO_x_ to the positively biased contact. The results in Figure 5 indicates that the effect of selective UV sensitivity also exists when electrons are injected from the top metal electrode into the SiO_x_ at negative gate voltage, as well as from the Si substrate at positive voltage (Figure 3). Further increase of responsivity may be obtained by optimization of the MOS structure design. For example, in transversal sensor the top electrode has to be semitransparent, while in lateral structure, in addition, comb-like contacts are more appropriate.

## 4. Conclusions

Selective UV sensitivity has been found in MOS structures with silicon nanocrystals in a SiO_2_ matrix and amorphous Si nanoparticles in a SiO_1.7_ matrix. I-V measurements in transversal and lateral configuration show that both types of structures have higher response to UV light than to light in the visible range. The UV selectivity is explained by assuming that light generates photocarriers in nanoclusters outside of the contact areas, which contribute to the current through the dielectric. The photogenerated carriers diffuse or are driven by the electric field under the top contacts leading to increase of the photocurrent. It was estimated that the material with a-Si nanoparticles responds to wavelengths close to or smaller than 480 nm, while the material with nanocrystals responds to wavelengths ≤ 690 nm. The observed difference results from the larger gap of the a-Si nanoparticles. The responsivity of the structure with nanocrystals to UV light is higher, which makes it attractive for UV sensor, though its response to visible light is also slightly higher. The top contacts of structures with Si nanocrystals have to be optimized in order to obtain higher responsivity when the material is used in UV sensors.
